# Immune-related intestinal pseudo-obstruction caused by immune checkpoint inhibitors: case report

**DOI:** 10.3389/fonc.2024.1415117

**Published:** 2024-08-14

**Authors:** Yimeng Qian, Zheng Zhi, Jing Ai, Lin Kang, Gang Qiu, Xin Huang, Jing Zhao

**Affiliations:** ^1^ Department of Oncology, Hebei General Hospital, Shijiazhuang, China; ^2^ Graduate School, Hebei North University, Zhangjiakou, China; ^3^ Department of Basic Medicine, Hebei University of Chinese Medicine, Shijiazhuang, China; ^4^ Graduate School, North China University of Science and Technology, Tangshan, China; ^5^ Department of Pathology, Hebei General Hospital, Shijiazhuang, China

**Keywords:** immune-related pseudo-obstruction, immune checkpoint inhibitor, adverse effects, bloating, constipation

## Abstract

Intestinal obstruction, a rare manifestation of immunotherapy-related gastrointestinal adverse events, can be severe and even life-threatening with intestinal perforation. We present a 64-year-old man with HCC and currently under the therapy with Pembrolizumab, who was admitted in our hospital with abdominal distension. Radiologic findings were consistent with small bowel ileus. After conservative treatment, the patient underwent colonoscopy where no cause of ileus was discovered. The patient received high-dose prednisone due to the side effects of immune checkpoint inhibitor therapy. This resulted in a gradual improvement of symptoms.

## 1 Introduction

Hepatocellular carcinoma (HCC) is a malignancy with high morbidity and mortality. The combination of immune checkpoint inhibitors (ICIs) and targeted therapy constitutes a pivotal approach in treating unresectable HCC. ICIs have positive effects. However, they can also cause (irAEs). These adverse reactions can affect nearly all organ systems in the body. The gastrointestinal tract is commonly involved, typically manifesting as diarrhea and colitis (1). However, intestinal obstruction as the primary manifestation of irAEs is rare. In this report, We present a case of rare intestinal pseudo-obstruction associated with pembrolizumab therapy, aiming to raise awareness of this rare irAE.

## 2 Case presentation

**Figure 1:**
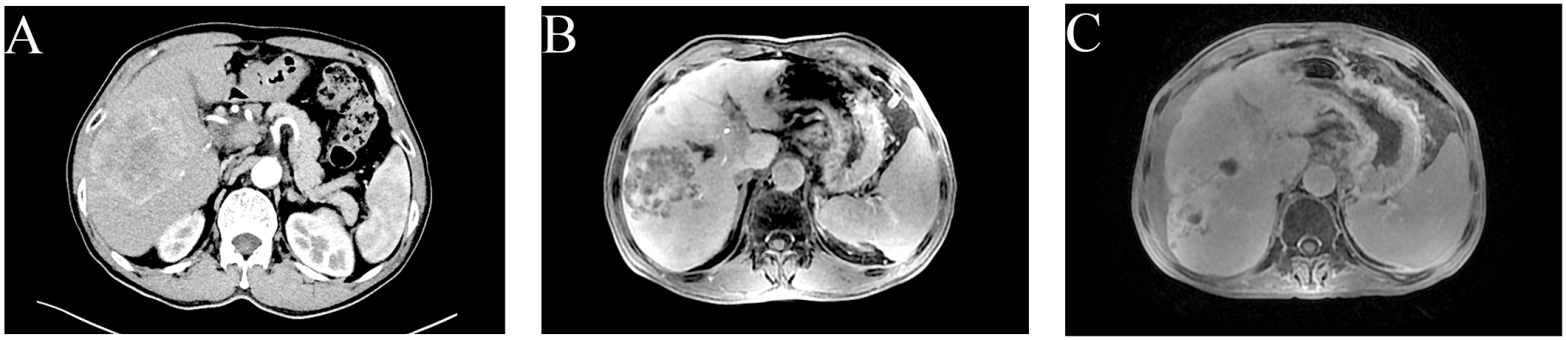
Clinical images of hepatocellular carcinoma in a 64-year-old man. **(A)** Advanced CT imaging has detected a significant mass in the right hepatic lobe, spanning multiple segments with dimensions of 112 mm x 77 mm x 83 mm. **(B)** Advanced CT imaging has identified a tumor in the right lobe of the liver with dimensions of 75 mm by 67 mm by 67 mm. Following completion of TACE, the efficacy of the treatment is PR in accordance mRECIST. **(C)** MRI revealed the largest hepatic tumor measuring approximately 69 mm x 63 mm x 67 mm. The efficacy of the radiotherapy has been evaluated as PR.

A 64-year-old male was admitted on January 19, 2023, presenting with pain localized to the right upper quadrant of the abdomen. An enhanced abdominal Computed Tomography (CT) scan revealed hepatocellular carcinoma (HCC) with portal vein thrombus, evidence of cirrhosis, and lymph node metastasis in the hepatic hilar and hepatogastric interstitial regions. The patient’s serum alpha fetoprotein (AFP) level was markedly elevated at 1210 ng/ml, exceeding the normal range of 0-7 ng/ml. The data from the Chinese population in the LEAP-002 study demonstrated a median overall survival (mOS) of 32.3 months and a median progression-free survival (mPFS) of 8.3 months in the lenvatinib combined with pembrolizumab group. These results confirm that the regimen of lenvatinib in combination with pembrolizumab provides a durable survival advantage for Chinese patients with advanced HCC (2). Consequently, on February 20, 2023, the patient initiated treatment with oral Lenvatinib at a dosage of 8 mg daily, in combination with pembrolizumab infusion at 200 mg administered every three weeks. Following two cycles of transarterial chemoembolization (TACE) with cisplatin (20 mg) and gemcitabine (2 g), a partial response was observed according to the modified Response Evaluation Criteria in Solid Tumors (mRECIST). Subsequent liver Magnetic Resonance Imaging (MRI) on April 27, 2023, demonstrated a reduction in tumor size within the right hepatic lobe, yet there was an increase in the extent of portal vein tumor thrombosis. Consequently, from June 1, 2023, to July 5, 2023, the patient underwent radiotherapy targeting the liver cancer and portal vein thrombosis. In the radiation therapy plan, the Gross Tumor Volume (GTV) encompasses lesions in segments V and VIII of the liver, while GTVth is defined as including thrombi within the portal vein and its branches. To accommodate uncertainties during treatment, such as positioning errors and organ movement, the GTV is expanded by 0.5 cm to form the Planning Gross Tumor Volume (PGTV), and the GTVth is similarly expanded to create the Planning Target Volume for thrombi (PTVth). Ninety-five percent of the PGTV volume receives a total dose of 4500 cGy, administered over 25 fractions; similarly, the PTVth’s 95% volume is treated with a total dose of 5800 cGy, also in 25 fractions. The terms PGTV and PTVth are specific to this treatment plan and are employed to distinguish between different treatment targets, whereas PTV typically signifies the Planning Target Volume. A follow-up CT scan conducted one month post-radiotherapy demonstrated continued partial response (PR) to treatment (**Figures 1A–C**).

**Figure 2:**
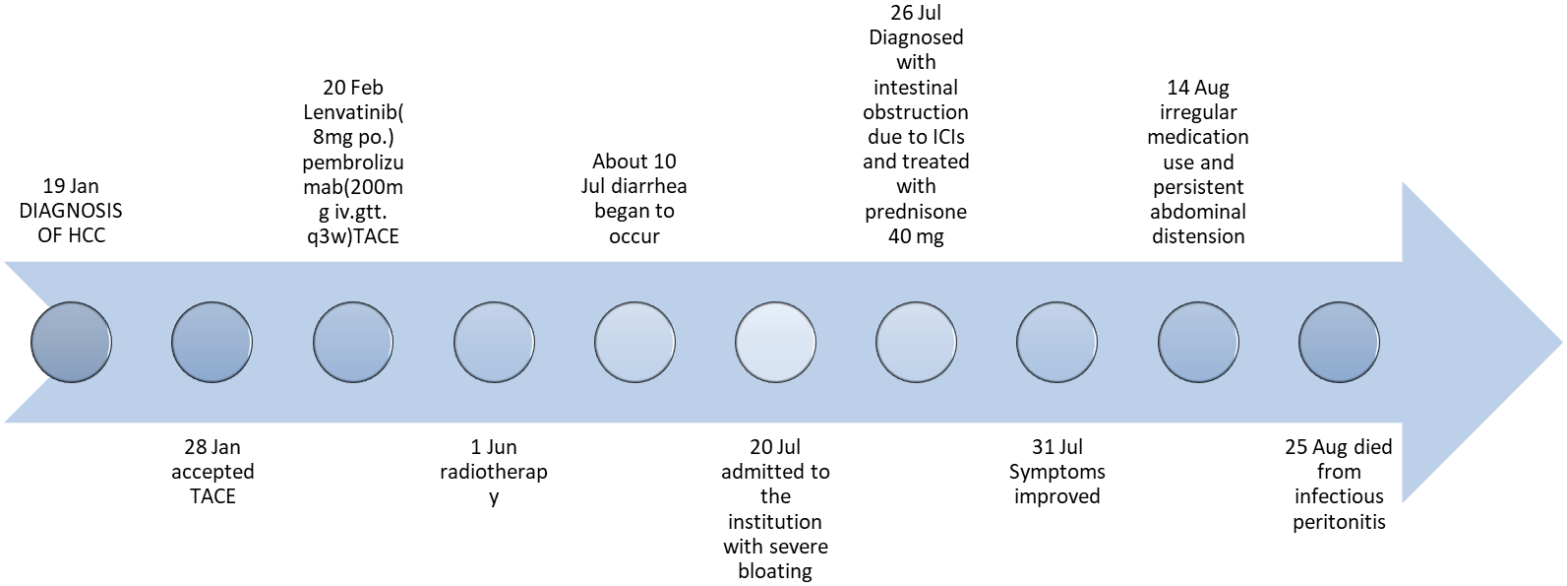
Timeline of the clinical course. TACE, transcatheter arterial chemoembolization; iv.gtt., intravenous drip; po., per os.

**Figure 3:**
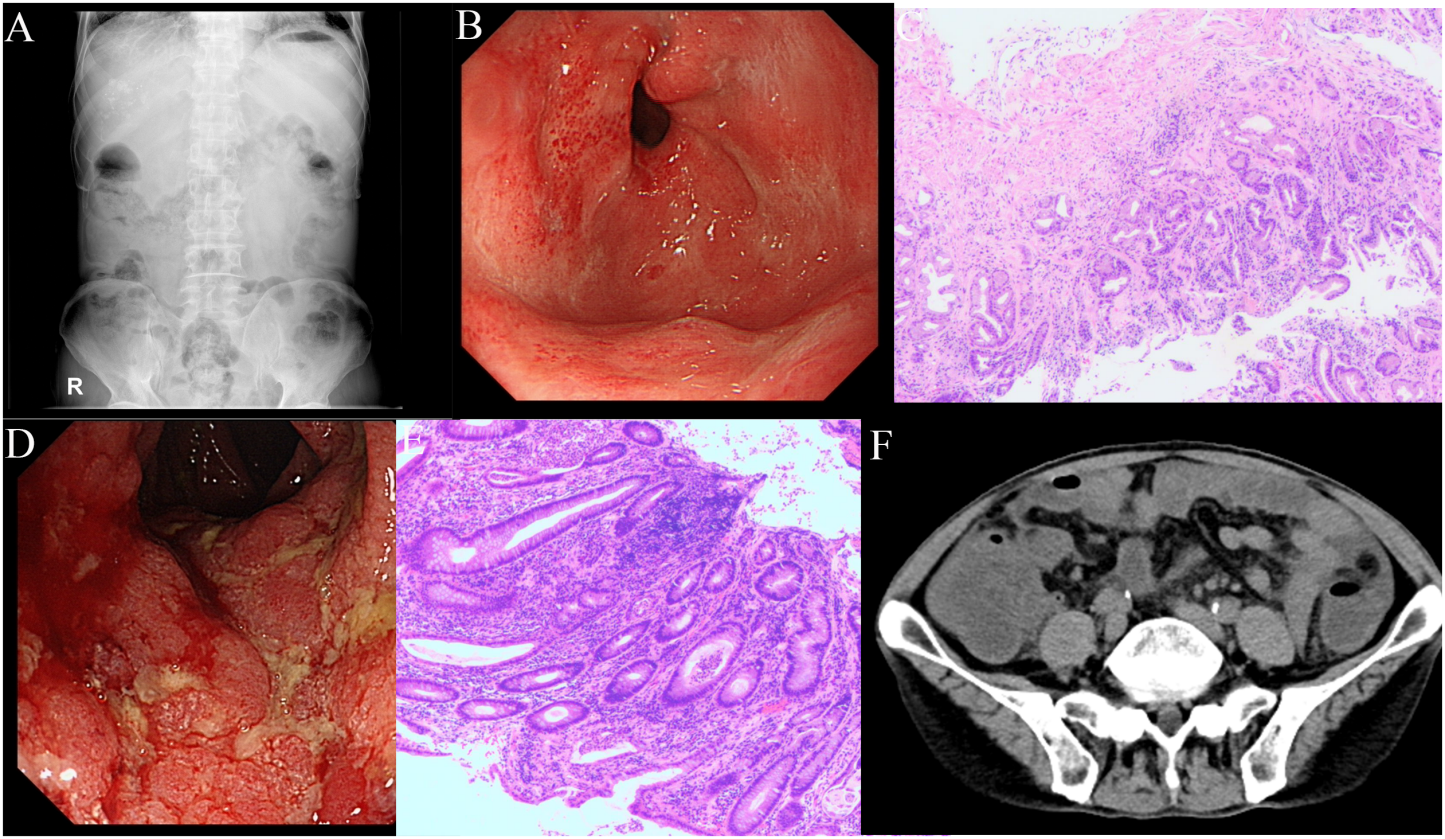
Clinical images during the treatment. **(A)** Plain abdominal x-ray shows no evidence of bowel distension or free intraperitoneal gas, but revealed increased colonic content and mild dilatation at the hepatic flexure of the right colon, along with gas-fluid levels. **(B)** Gastroscopy demonstrated features of chronic non-atrophic gastritis, with congested and edematous gastric folds, multiple ulcerative scars in the antrum, and flaky congestion in the duodenal papilla. **(C)** Pathological examination confirmed mild chronic mucosal inflammation with interstitial vascular congestion and a minor eosinophilic infiltrate. **(D)** Colonoscopy revealed congestion and edema of the colorectal mucosa, indistinct vascular patterns, friable mucosa prone to contact bleeding, and superficial erosions and ulcerations, leading to a diagnosis of colitis. **(E)** The pathology indicates a moderate level of chronic inflammation in the mucosa, with the presence of cryptitis and crypt abscesses. **(F)** Abdominal and pelvic scans revealed colonic dilation with fluid and gas accumulation.

Following six cycles of immune checkpoint inhibitor (ICI) therapy, the patient developed diarrhea, with episodes up to four times daily. **Figure 2** provides a comprehensive illustration of the patient's detailed treatment process.This was managed with montmorillonite powder for toxin absorption and loperamide to regulate bowel movements. However, diarrhea recurred upon cessation of these medications. On July 20, 2023, the patient presented with constipation and bloating, initially considered non-severe but later necessitating hospitalization due to worsening symptoms. Physical examination indicated abdominal tenderness without signs of peritoneal irritation and hypoactive bowel sounds. After a Multi-Disciplinary Treatment (MDT) discussion, the patient was prescribed metoclopramide, neostigmine, and simethicone, aiming to enhance gastrointestinal motility and alleviate symptoms. Despite these interventions, the patient’s response was suboptimal. Abdominal radiography showed no evidence of bowel distension or free intraperitoneal gas, but revealed increased colonic content and mild dilatation at the hepatic flexure of the right colon, along with gas-fluid levels (**Figure 3A**). Gastroscopy demonstrated features of chronic non-atrophic gastritis (**Figure 3B**). Pathological examination confirmed mild chronic mucosal inflammation with interstitial vascular congestion and a minor eosinophilic infiltrate (**Figure 3C**). Colonoscopy revealed the presence of colitis (**Figure 3D**). Histopathology confirmed moderate chronic mucosal inflammation with cryptitis and crypt abscess formation (**Figure 3E**). Further imaging with abdominal and pelvic scans showed colonic dilation with fluid and gas, but no intraluminal stenosis, suggesting functional obstruction or inflammatory disease without mechanical blockage (**Figure 3F**). Having ruled out infection, tumor progression and other possible factors, the patient was diagnosed with immunotherapy-related bowel obstruction and started on 40mg of prednisone, which resulted in a significant improvement in abdominal distension within five days. Upon discharge, the patient was advised to continue oral prednisone. However, inconsistent medication adherence post-discharge was associated with persistent, albeit improved, abdominal distension. In August 2023, the patient was readmitted with acute abdominal pain and distension, indicative of a severe complication. Despite aggressive therapeutic interventions, the patient tragically succumbed to infectious peritonitis.

## 3 Discussion

Intestinal obstruction represents a rare manifestation of gastrointestinal irAEs (GI irAEs) (3). The onset of intestinal obstruction varies widely, posing a significant challenge for physicians in determining the precise onset time. Out of the seven reported cases (4–9), four showed that intestinal obstruction developed late in the treatment course after the initiation of ICIs (11 cycles of pembrolizumab, 14 cycles of nivolumab, 8 cycles of pembrolizumab, and 14 cycles of nivolumab, respectively). Two patients (9) presented with bowel obstruction shortly after starting ICIs. One patient received two cycles of ipilimumab, while the other received one cycle of nivolumab in combination with ipilimumab. Furthermore, a single patient experienced abdominal distension after receiving one cycle of nivolumab in combination with ipilimumab. Pseudo-obstruction frequently manifests rapidly following ICI treatment, although the precise timing is rarely specified in the literature. Only a subset of the literature provides an approximate time frame, with Appelbaum’s case (9) being described as “rapid” for symptom onset and Ishibashi’s case (4) as “acute”. The lack of specific data points makes it challenging to quantify the exact time of onset of intestinal obstruction.

ICIs are monoclonal antibodies that target the cytotoxic T-lymphocyte-associated protein 4 (CTLA-4) and the programmed death-1 (PD-1)/programmed death-ligand 1 (PD-L1) pathways (10). They have transformed cancer treatment. These drugs enhance the anti-tumor T-cell response and may cause gastrointestinal infections. Unlike traditional cytotoxic drugs, the mechanism behind gastrointestinal irAEs is thought to involve an imbalance between effector T-cells and regulatory T-cells (Tregs), which can lead to Treg depletion or dysfunction (11). GI irAEs are driven by multiple immunological and molecular mechanisms, including autoantibody production (12), molecular mimicry (13), cytokine production (14), and influences from the gut microbiome (15) and genetic factors (16). Patients with pseudo-obstruction typically exhibit severe chronic “obstructive” symptoms such as abdominal pain, distention, bloating, nausea, vomiting, diarrhea, and/or refractory constipation. A case report described a patient undergoing treatment with nivolumab for advanced non-small cell lung cancer experienced severe abdominal pain with diffuse abdominal tenderness, vomiting, and hypotension (5). After exclusionary diagnosis, it was determined that the patient had small bowel obstruction with colonic perforation caused by ICIs. Similarly, there have been reports of patients receiving a combination of PD-L1 monoclonal antibody atezolizumab with etoposide and carboplatin for advanced small cell lung cancer, presenting with symptoms of abdominal distension, diarrhea, and constipation, ultimately leading to consideration of immune therapy-related intestinal obstruction (8).

Intestinal obstruction associated with ICIs is a non-specific condition that requires an exclusionary diagnosis to identify. Before identifying immunotherapy-associated bowel obstruction, it is important to rule out infection, potential progression of malignancy, and other possible factors. The examination should comprise diagnostic imaging, laboratory tests, and pathogen cultures that are specific to the patient’s symptoms (5). Colonoscopy typically reveals congestion and edema of the bowel mucosa. The potential for bowel perforation should be communicated to the patient. Exploratory surgery should be performed for pathological diagnosis if necessary. Pathological examination may reveal chronic inflammation in the colon, characterized by crypt and glandular abnormalities, along with infiltration of lymphocytes and plasma cells in the lamina propria, and epithelial cell apoptosis. However, these findings lack specificity, making it difficult to diagnose intestinal obstruction caused by ICIs.

**Figure 4:**
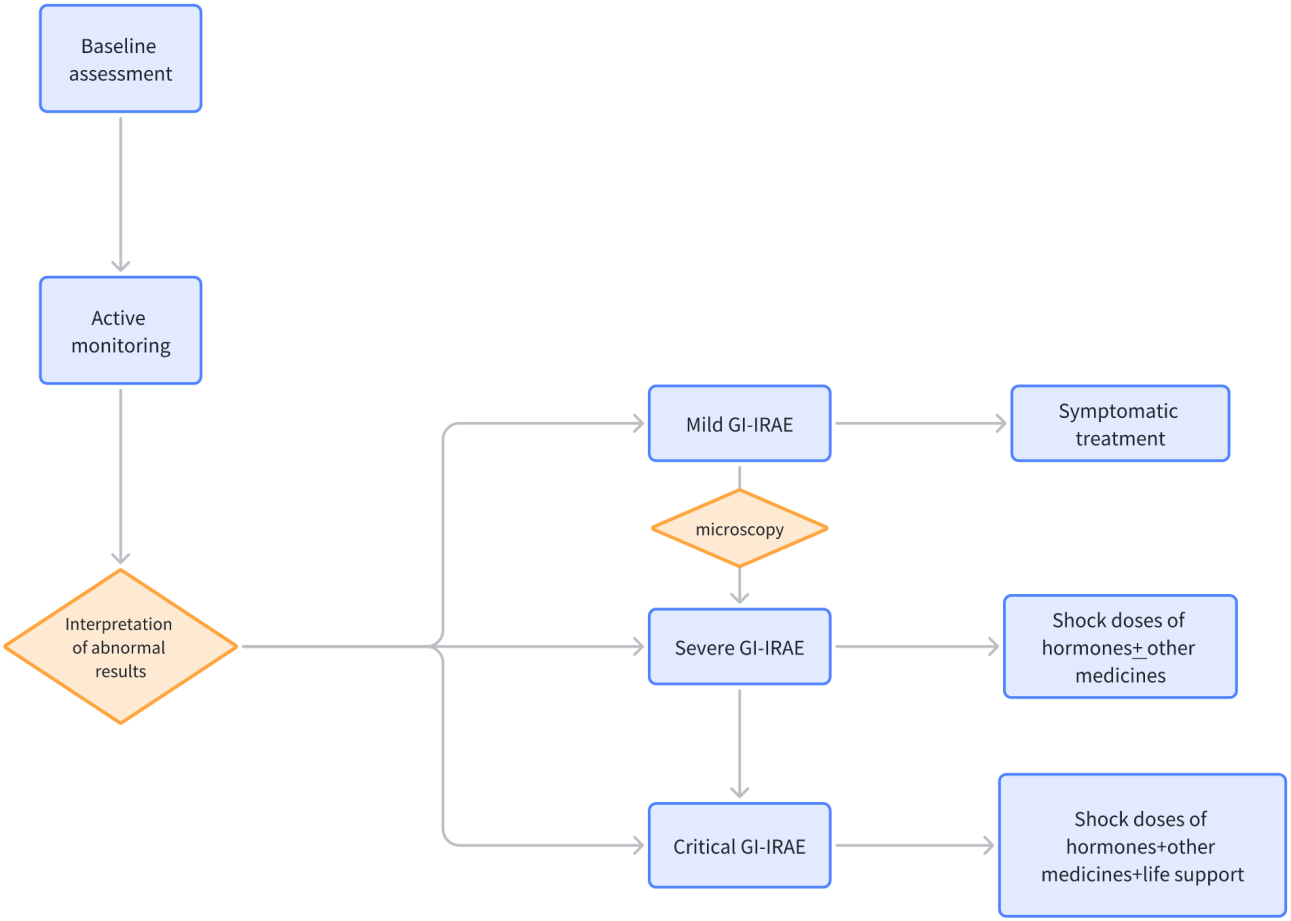
GI irAEs Management Flowchart.

Due to the low incidence, there is a lack of systemic experience in managing pseudo-intestinal obstruction. Based on available case reports, patients diagnosed with immunotherapy-associated intestinal obstruction are advised to first suspend the use of ICIs. For patients presenting with symptoms of GI, such as diarrhea and abdominal pain, detailed clinical assessments and necessary laboratory investigations are required, including routine blood, liver and kidney functions, electrolytes, stool routines, and cultures. In the case of mild symptoms, symptomatic treatment may be sufficient, comprising the use of antidiarrheal medication (e.g., loperamide) and maintenance of appropriate electrolyte and fluid balance. For moderate to severe GI irAEs, systemic corticosteroid therapy, such as oral prednisone at a dosage of 0.5-1 mg/kg/day, is typically indicated. In cases of greater severity, intravenous methylprednisolone at a dosage of 1-2 mg/kg/day may be necessary (17). Should corticosteroid treatment prove ineffective or symptoms fail to improve, additional immunosuppressive agents, such as infliximab, mycophenolate mofetil, or tacrolimus, may be required. In refractory cases, biological agents such as anti-TNF-α antibodies may be required (18). In cases of moderate to severe severity, endoscopic procedures such as colonoscopy or gastroscopy may be required to assess the condition of the gastrointestinal mucosa and to obtain tissue samples for pathological examination. Should the presence of infectious complications be detected, targeted antibiotics are required. Nutritional support, including enteral or parenteral nutrition, may be necessary during treatment. **Figure 4** illustrates the management flowchart for GI irAEs. The treatment of GI irAEs requires requires a multidisciplinary team, including oncology, gastroenterology, and nutrition. It is important to provide patients and their families with information about GI irAEs, including possible symptoms, treatment options, and precautions. It is also essential to emphasize that GI irAEs require long-term follow-up and management, and even after symptom resolution, regular follow-up is needed to monitor for potential recurrence. It is of utmost importance that treatment is initiated promptly to minimize the potential for complications and to improve the patient’s prognosis.

## 4 Conclusion

In conclusion, this case highlights a unique presentation of functional intestinal pseudo-obstruction in a patient with HCC treated with the PD-1 inhibitor pembrolizumab. Early diagnosis of pseudo-intestinal obstruction can be challenging. Clinicians should be aware that early identification and intervention can improve prognosis without compromising immunotherapy. However, severe conditions require the suspension of ICIs. If the condition is severe, suspend ICIs and use corticosteroids or other immunosuppressants, depending on the condition. ICIs are commonly used in clinical settings, but they can cause irAEs, which is a cause for concern. The management of gastrointestinal irAEs requires close monitoring of patients after administration of the drug. It is important to promptly identify and address relevant symptoms.

## Data Availability

The original contributions presented in the study are included in the article/supplementary material. Further inquiries can be directed to the corresponding author.
